# 重组人血管内皮抑素联合化疗与单纯化疗在晚期非小细胞肺癌中疗效比较的系统评价

**DOI:** 10.3779/j.issn.1009-3419.2011.05.05

**Published:** 2011-05-20

**Authors:** 德东 曹, 伟 戈, 慧敏 王, 令 张, 永法 郑, 金忠 张

**Affiliations:** 430060 武汉，武汉大学人民医院肿瘤科 Department of Medical Oncology, Remmin Hospital of Wuhan University, Wuhan 430060, China

**Keywords:** 肺肿瘤, 化疗, 重组人血管内皮抑素, 系统评价, Lung neoplasms, Chemotherapy, rh-endostatin, Systematic review

## Abstract

**背景与目的:**

近年来已有大量有关抗血管生成药物重组人血管内皮抑素（恩度，rh-endostatin）在肺癌治疗中有效性和安全性的研究与报道，真实评价重组人血管内皮抑素对肺癌的治疗效果具有重要意义，本文将系统评价重组人血管内皮抑素联合化疗与单纯化疗在晚期非小细胞肺癌（non-small cell lung cancer, NSCLC）治疗中的疗效与安全性。

**方法:**

采用Cochrane系统评价方法，对Embase、Medline、SCI、Cochrane图书馆、中国生物医学文献数据库、中文科技期刊全文数据库、中国期刊全文数据库等数据库进行计算机检索，检索时间截止至2010年3月。纳入标准为研究对象为晚期NSCLC病例，实验组为重组人血管内皮抑素联合化疗，对照组为单独使用化疗，研究内容为比较两组治疗疗效的随机对照试验。纳入研究的质量由两名研究者评估。采用RevMan 5.0软件对研究进行*meta*分析。

**结果:**

按照标准共纳入15篇文献，1, 326例病例。15项研究均为临床随机对照试验，其中2项对随机方法进行了详细描述，13项未对盲法进行描述。*Meta*分析表明，长春瑞滨+顺铂+重组人血管内皮抑素方案（NPE）与长春瑞滨+顺铂方案（NP）比较反应率差异有统计学意义（OR=2.16, 95%CI: 1.57-2.99），NPE方案治疗后重度白细胞减少（OR=0.94, 95%CI: 0.66-1.32）、重度血小板下降（OR=1.00, 95%CI: 0.64-1.57）和恶心呕吐（OR=0.85, 95%CI: 0.61-1.20）发生率均与NP方案比较无显著性差异（*P* > 0.05）。NPE+放疗方案与NP+放疗方案的反应率相似（OR=2.39, 95%CI:0.99-5.79），NPE+放疗方案治疗后白细胞减少（OR=0.83, 95%CI: 0.35-1.94）、血小板下降（OR=0.78, 95%CI: 0.19-3.16）和放射性食管炎（OR=1.00, 95%CI: 0.40-2.49）发生率均与NP+放疗方案比较无显著性差异（*P* > 0.05）。

**结论:**

在晚期NSCLC的治疗中，重组人血管内皮抑素与以铂类为基础的化疗方案联用可提高治疗反应率，同时毒副作用增加不明显，但在此基础上再与放疗联用并不能提高治疗反应率。由于本文纳入的研究较少，发生偏倚可能性较大，从而影响到结论的论证强度，尚需更多高质量的双盲随机对照研究提供证据。

## Abstract (en)

**Background and objective:**

In recent years, there has been a large number of studies and reports about the efficacy and safety of recombinant human endostatin (rh-endostatin), an anti-angiogenic drug, in treatment of advanced lung cancer. Authentic assessment of rh-endostatin treatment in lung cancer is important. The aim of this study is to assess the clinical efficacy and safety of rh-endostatin combined with chemotherapy in the treatment of patients with non-small cell lung cancer (NSCLC).

**Methods:**

Cochrane systematic review methods were used in the data selection, and data were selected from the Cochrane Library, EMBASE, Medline, SCI, CBM, CNKI, and etc electronic database to get all clinical controlled trials. The retrieval time was March 2010. The objects of these randomized controlled trials were advanced NSCLC patients and in the experimental group was rh-endostatin combination chemotherapy, in the control group was chemotherapy alone to compare the efficacy of two groups. The quality of included trials were evaluated by two reviewers independently. The software RevMan 5.0 was used for *meta*-analyses.

**Results:**

Fifteen trials with 1, 326 patients were included according to the including criterion. All trials were randomized controlled trials, and two trials were adequate in reporting randomization. Thirteen trials didn't mention the blinding methods. *Meta* analysis indicated that the NPE arm (Vinorelbine+cisplatin+rh-endostatin) had a different response rate compared with NP (Vinorelbine+cisplatin) arm (OR=2.16, 95%CI: 1.57-2.99). The incidences of severe leukopenia (OR=0.94, 95%CI: 0.66-1.32) and severe thrombocytopenia (OR=1.00, 95%CI: 0.64-1.57) and nausea and vomiting (OR=0.85, 95%CI: 0.61-1.20) were similar in the NPE arm compared with those in the NP arm. The NPE plus radiotherapy (RT) arm had a similar response rate compared with NP plus RT arm (OR=2.39, 95%CI: 0.99-5.79). The incidences of leukopenia (OR=0.83, 95%CI: 0.35-1.94) and thrombocytopenia (OR=0.78, 95%CI: 0.19-3.16) and radiation esophagitis (OR=1.00, 95%CI: 0.40-2.49) were similar in the NPE plus RT arm compared with those in the NP plus RT arm.

**Conclusion:**

In the treatment of advanced NSCLC, rh-endostatin in combination with platinum-based chemotherapy improve the response rate without obviously raised side effects, however, when radiotherapy are added to NPE arm or NP arm, the response rates have a similar outcome. Owing to the small sample size and poor quality of included trials, more well-designed double-blinded randomized controlled trials should be performed.

肺癌由于其发生率高且预后差，目前仍是威胁人类公共健康的主要疾病之一。非小细胞肺癌（non-small cell lung cancer, NSCLC）占所有肺部肿瘤的80%以上^[1]^。对于早期的NSCLC手术切除是首选治疗方法，但是大约有70%的患者就诊时被诊断为晚期NSCLC（局部晚期或出现转移病灶）^[2]^。由于晚期NSCLC具有病灶相对较大且易转移的特性，使以铂类等药物为基础的化疗疗效不佳且易发生并发症。

肿瘤抗血管生成是目前研究的热点之一，重组人血管内皮抑素（商品名：恩度，rh-endostatin）是众多抗血管生成药物之一。1997年O’Reilly发现内皮抑素，并发现内皮抑素是一种内源性抗血管生成物质，它通过作用于内皮细胞，尤其是微血管内皮细胞，从而达到阻止内皮细胞迁移并诱导其凋亡的作用^[3]^，同时也具有抑制血管内皮生长因子、抑制金属蛋白酶、与肝素样硫酸蛋白结合、与锌离子结合、影响诸如*HIF-1α*等基因表达的作用^[4]^。近年来，重组人血管内皮抑素联合常规细胞毒疗法用于治疗肿瘤的体内外研究日益增多，并且研究显示联合疗法疗效优于常规疗法疗效^[5, 6]^。但是抗血管生成疗法联合化疗是否真正可以使晚期NSCLC患者受益，联合应用时的安全性如何等方面的问题文献报道不一，因此本文通过对国内外主要电子数据库进行检索，筛选出符合要求的随机对照试验证据并进行系统分析，以期为重组人血管内皮抑素联合化疗治疗晚期NSCLC的临床应用提供疗效、安全性及毒副作用方面的循证依据。

## 1 资料与方法

### 1.1 纳入标准

#### 1.1.1 研究对象

① 经细胞学或病理学诊断为NSCLC；②影像学或其它临床检查确定为Ⅲ期、Ⅳ期NSCLC；③无年龄、性别限制；④治疗前血常规、尿常规、肝肾功等检查未见明显异常者。

#### 1.1.2 设计类型

重组人血管内皮抑素联合常规化疗治疗晚期NSCLC的随机对照试验，试验设置平行对照，样本例数在40例及以上。

#### 1.1.3 干预措施

① 重组人血管内皮抑素+化疗方案A *vs*化疗方案A；②重组人血管内皮抑素替代化疗方案A中一种或多种药物*vs*化疗方案A；③重组人血管内皮抑素+化疗方案A *vs*化疗方案B；④重组人血管内皮抑素+化疗方案A+放疗*vs*化疗方案A+放疗。

#### 1.1.4 疗效判定指标

① 总体生存率；②中位疾病进展期；③中位生存期；④有效率；⑤生存质量；⑥不良反应（按WHO毒副反应标准）。

### 1.2 排除标准

① NSCLC为转移性肺癌；②同时患有其它肿瘤疾病；③存在严重内科疾患及感染；④选入的研究与上述纳入标准不符；⑤无法验证文章真实性：主要指验证研究中原始信息的正确性和一致性。

### 1.3 检索策略

所用英文检索词为：non small cell lung cancer、non small cell lung、carcinoma、lung alveolus cell carcinoma、lung adenocarcinoma、NSCLC；drug therapy、antiangiogenic、antiangiogenesis、adjuvant therapy、combination therapy、endostatin、rh-endostatin、chemotherapy。中文检索词为：化疗、恩度、内皮抑素、重组人血管内皮抑素、抗血管生成治疗、靶向药物、肿瘤血管、肺癌、肺肿瘤、非小细胞肺癌。文献类型为：systematic reviews、*meta*-analyses、major clinical studies、randomized controlled trials (RCT)、practice guidelines。

#### 1.3.1 计算机检索

Embase（1980年-2010年）、Medline（1966年-2010年3月）、SCI（1974年-2010年）、Cochrane图书馆（1998年-2009年）、中国生物医学文献数据库（1989年-2010年）、中文科技期刊全文数据库（1989年-2010年）、中国期刊全文数据库（1994年-2010年）。检索文献为中文或英文。

#### 1.3.2 手工检索

在图书馆检索杂志：lung cancer、cancer、中国肺癌杂志、中华肿瘤杂志、癌症、肿瘤、中国肿瘤临床、实用肿瘤杂志。年限为1995年-2009年。

#### 1.3.3 其它检索

利用Google搜索互联网上相关文献及其参考文献，与作者联系，追踪详细数据或原文等。

### 1.4 试验筛选

由作者本人独立选择研究，按设计的表格填写资料，由两名研究者对纳入研究的质量进行评估。当有问题时与他人讨论解决，无法通过数据库获得的资料尽量与作者联系，保证资料齐全。

### 1.5 方法学质量评价

所用质量评价为Cochrane图书馆系统评价手册5，评价质量主要涉及以下几个方面：①随机方法，根据是否采用随机方法且其使用方法是否合理可分为三类：正确和充分、不充分、不清楚；②隐蔽分组，分为四类：正确和充分、不充分、不清楚和未使用；③盲法，是否合理采用盲法，可分为单盲、双盲和三盲；④失访及其处理，有无全程随访、报告失访人数、失访人数是否不超过10%及有没有采用意向性处理分析。

研究质量可分为A、B、C三级：A级：轻度偏倚，完全符合以上质量标准，各种偏倚发生的可能性最小；B级：中度偏倚，部分满足其中一条或更多标准，偏倚发生的可能性中度；C级：高度偏倚，完全不满足的任何一条或更多标准，偏倚发生可能性最高。

### 1.6 资料提取

提取的资料有如下几种：①一般资料，包括试验的题目、作者、出版日期、文献来源；②试验特征，包括试验设计、研究和随访时间、干预措施、测量指标、失访人数及处理；③结局指标，包括反应率、生存率、症状改善、不良反应等。

### 1.7 统计学处理

使用Cechrane协作网提供的RevMan 5.0软件进行*meta*分析。计数资料采用相对危险度（relative risk, RR）或优势比（odd ratio, OR）及其95%可信区间（confidence intervals, CI）表示，计量资料计算权重均差WMD及相应95%可信区间表示。采用卡方检验对各亚组试验结果之间的异质性进行分析（检验水准α=0.1），当亚组内各研究之间的资料相似性充足时（*I*^2^ < 50%, *P* > 0.1）应用固定效应模型（fixed effects model）合并分析，反之使用随机效应模型（random effects model）。如各研究之间无临床同质性时分别进行描述。

## 2 结果

### 2.1 纳入研究的基本情况

通过对前述数据库及相关杂志进行检索，初步筛选出62篇文献，其中中文48篇，英文12篇。通过进一步阅读文献排除非随机对照和重复研究后有21篇文献^[7-27]^为临床随机对照研究，且全为中文。排除文献6篇^[17, 19, 24-27]^，其中3篇文献^[24-26]^的样本例数不满足纳入要求，2篇文献^[17, 19]^研究干预措施不符合要求，1篇文献^[27]^无法验证文章真实性，最终纳入文献15篇^[7-16, 18, 20-23]^。

#### 2.1.1 研究设计

本文所纳入的15篇文献中共包含15项研究，均为临床随机对照研究，其中2项研究采用数字表随机方法^[10, 20]^，其余未报道具体随机方法^[7-9, 11-16, 18, 21-23]^。15项研究均未对随机方案的隐藏进行详细报道。2项研究采用盲法^[18, 20]^，1项研究未采用盲法^[15]^，其余12项研究均未报道是否采用盲法^[7-14, 16, 21-23]^。有3项研究报道失访^[18, 20, 23]^，1项研究在完成治疗后进行了随访^[21]^。按照Cochrane系统评价员手册5.0版的推荐标准对所纳入研究进行质量评价，均有不同程度的偏倚发生，结果见表 1。

**1:** 纳入研究的质量分析 Quality analysis of included studies

Included study	Randomized method	Allocation hidden	Blind	Lost	Quality of study
Yang L^[23]^	unclear	insufficient	unclear	2 cases	C
Wang JW^[20]^	sufficient	insufficient	clear	7 cases	B
Huang C^[18]^	clear	sufficient	clear	7 cases	B
Chen SJ^[10]^	clear	insufficient	unclear	not reported	B
Huang GS^[7]^	unclear	insufficient	unclear	not reported	C
Fan QL^[22]^	unclear	insufficient	unclear	not reported	C
Cai L^[13]^	unclear	insufficient	unclear	not reported	C
Xie YR^[9]^	clear	insufficient	unclear	not reported	B
Liu J^[16]^	unclear	insufficient	unclear	not reported	B
Ma JB^[21]^	unclear	insufficient	unclear	not reported	C
Zhang T^[14]^	unclear	insufficient	unclear	not reported	C
Tang Z^[8]^	unclear	insufficient	unclear	not reported	C
Han LC^[11]^	unclear	insufficient	unclear	not reported	C
Jin J^[12]^	unclear	insufficient	unclear	not reported	C
Liu J^[15]^	unclear	insufficient	unclear	not reported	C

#### 2.1.2 研究对象

共纳入晚期NSCLC病例1, 326例，全部为中国患者，其中男性923例，女性403例，Ⅲ期患者682例，Ⅳ期患者644例，纳入研究的基本情况见表 2。

**2:** 纳入研究临床实验项目基本资料 Basic information of included clinical studies

Study	Region	Time (year)	Grade	Sample	Experimental group (case)	Control group (case)	Standard methods of quality of life
Yang L^[23]^	Multi-center	2002-2003	Ⅲ, Ⅳ	87	54	33	ECGO
Wang JW^[20]^	Multi-center	2003-2004	Ⅲ, Ⅳ	486	322	164	ECGO
Huang C^[10]^	Tianjin	2005	Ⅲ, Ⅳ	74	50	24	none
Chen SJ^[10]^	Guangxi	2005-2007	Ⅳ	50	24	26	ECGO
Huang GS^[7]^	Henan	2006-2007	Ⅲ, Ⅳ	40	20	20	karnofsky
Fan QL^[22]^	Shandong	2006-2007	Ⅲ, Ⅳ	40	20	20	karnofsky
Cai L^[13]^	Heilongjiang	2006-2007	Ⅲ, Ⅳ	71	39	32	karnofsky
Xie YR^[9]^	Zhejiang	2006-2008	Ⅲ, Ⅳ	48	22	26	karnofsky
Liu J^[16]^	Jiin	2007-2008	Ⅲ, Ⅳ	62	31	31	karnofsky
Ma JB^[21]^	Sichuan	2007-2008	Ⅲ	46	23	23	ECGO
Zhang T^[14]^	Zhejiang	2007-2008	Ⅲ, Ⅳ	104	48	56	karnofsky
Tang Z^[8]^	Guangdong	2007-2008	Ⅲ, Ⅳ	53	27	26	karnofsky
Han LC^[11]^	Jiin	2007-2009	Ⅲ, Ⅳ	68	37	31	none
Jin J^[12]^	Hunan	2008	Ⅲ	40	15	25	none
Liu J^[15]^	Henan	2008-2009	Ⅲ, Ⅳ	60	30	30	karnofsky

#### 2.1.3 干预措施

所纳入的研究均采用静脉途径给药。其中7项研究^[7, 12, 13, 18, 20, 22, 23]^进行了长春瑞滨+顺铂+重组人血管内皮抑素（NPE）方案与长春瑞滨+顺铂（NP）方案的比较，2项研究^[16, 21]^进行了NPE+放疗方案与NP+放疗方案的比较，1项研究^[10]^进行了长春瑞滨+奥沙利铂+重组人血管内皮抑素（NOE）方案与长春瑞滨+奥沙利铂（NO）方案的比较，1项研究^[8]^进行了紫杉醇+卡铂+重组人血管内皮抑素（TCE）方案与紫杉醇+卡铂（TC）方案的比较，1项研究^[9]^进行了吉西他滨+顺铂+重组人血管内皮抑素（GPE）方案与吉西他滨+顺铂（GP）方案的比较，1项研究^[14]^进行了GPE方案与紫杉醇（T）方案的比较，1项研究^[15]^进行了紫杉醇+重组人血管内皮抑素（TE）方案与T方案的比较，1项研究^[11]^进行了紫杉醇+顺铂+重组人血管内皮抑素（TPE）方案与紫杉醇+顺铂（TP）方案的比较，具体情况见表 3。

**3:** 纳入研究的干预措施及结局指标 Inventions and endpoint of included trials

Study	Invention group			Case		Endpoint
	Experimental	Control		Experimental	Control	
Yang L^[23]^	NPE	NP		54	33	RR, SI, Adverse reactions
Wang JW^[20]^	NPE	NP+placebo		322	164	RR, SI, Adverse reactions
Huang C^[18]^	NPE	NP		50	24	RR, Survival rate, Adverse reactions
Chen SJ^[10]^	NOE	NO		24	26	RR, Adverse reactions
Huang GS^[7]^	NPE	NP		20	20	RR, Survival rate, SI, Adverse reactions
Fan QL^[22]^	NPE	NP		20	20	RR, Adverse reactions
Cai L^[13]^	NPE	NP		39	32	RR, Survival rate, SI, Adverse reactions
XieYR^[9]^	GPE	GP		22	26	RR, Survival rate, SI, Adverse reactions
Liu J^[16]^	NPE+RT	NP+RT		31	31	RR, Survival rate, Adverse reactions
Ma JB^[21]^	NPE+RT	NP+RT		23	23	RR, Survival rate, SI, Adverse reactions
Zhang T^[14]^	GPE	T		48	56	RR, SI, Adverse reactions
Tang Z^[8]^	TCE	TC		27	26	RR, Adverse reactions
Han LC^[11]^	TPE	TP		37	31	RR, Adverse reactions
Jin J^[12]^	NPE	NP		15	25	RR, Survival rate, Adverse reactions
Liu J^[15]^	TE	T		30	30	RR, Adverse reactions
NPE: Vinorelbine+Oxaliplatin+rh-endostatin; NOE: Vinorelbine+Oxaliplatin+rh-endostatin; GPE: Gemcitabine+Cisplatin+rh-endostatin; TCE: Paclitaxel+Carboplatin+rh-endostatin; NP: Vinorelbine+Oxaliplatin; NO: Vinorelbine+Oxaliplatin; GP: Gemcitabine+Cisplatin; TC: Paclitaxel +Carboplatin; T: Paclitaxel; RT: radiotherapy; RR: response rate; SI：symptom improvement.								

#### 2.1.4 结局指标

纳入的15项研究均对反应率RR（PR+CR）进行了报道，14项研究^[7-16, 20-23]^报道了毒副反应，1项研究^[21]^报道了无进展生存率和1年生存率，7项研究^[9, 10, 12, 16, 18, 20, 23]^报道了中位疾病进展期，3项研究^[7, 9, 14]^报道了karnofsky标准下的体力改善。

### 2.2 *meta*分析结果

#### 2.2.1 客观反应率

##### 2.2.1.1 NPE方案与NP方案

7项研究^[7, 12, 13, 18, 20, 22, 23]^报道了治疗后反应率，分析结果显示NPE方案与NP方案的反应率差异有统计学意义（OR=2.16, 95%CI: 1.57-2.99, *P* < 0.05）（图 1）。

**1:**
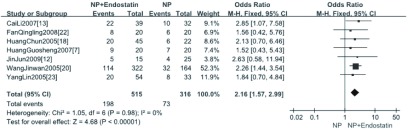
NP+重组人血管内皮抑素方案与NP方案治疗后反应率的*meta*分析 *Meta* -analysis of the RR between NP plus rh-endostatin and NP alone

##### 2.2.1.2 NPE+放疗方案与NP+放疗方案

2项研究^[16, 21]^报道了治疗后反应率，分析结果显示NPE+放疗（RT）方案与NP+放疗方案的反应率差异无统计学意义（OR=2.39, 95%CI: 0.99-5.79）（图 2）。

**2:**
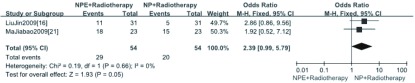
NPE+放疗方案与NP+放疗方案治疗后反应率的*meta*分析 *Meta*-analysis of the RR between NPE+RT and NP+RT scheme

#### 2.2.2 毒副反应

##### 2.2.2.1 白细胞下降

5项研究^[7, 12, 13, 20, 23]^报道了NPE方案与NP方案相比治疗后重度（WHO标准Ⅲ级、Ⅳ级）白细胞下降的情况，分析结果显示NPE方案治疗后重度白细胞下降与NP方案相比差异无统计学意义（OR=0.94, 95%CI: 0.66-1.32, *P* > 0.05）（图 3）。2项研究^[16, 21]^报道了NPE+放疗方案与NP+放疗方案相比白细胞下降的情况，分析结果显示NPE+放疗方案治疗后白细胞下降与NP+放疗方案相比较差异无统计学意义（OR=0.83, 95%CI: 0.35-1.94, *P* > 0.05）（图 4）。

**3:**
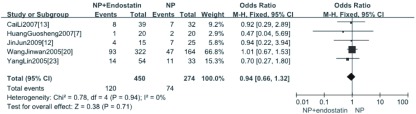
NP+重组人血管内皮抑素方案与NP方案治疗后重度白细胞下降*meta*分析 *Meta*-analysis of the severe leucopenia between NPE and NP alone

**4:**
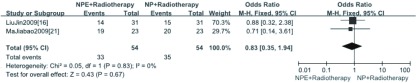
NPE+放疗方案与NP+放疗方案治疗后白细胞下降的*meta*分析 *Meta*-analysis of the leucopenia between NPE+RT and NP+RT

##### 2.2.2.2 血小板下降

3项研究^[13, 20, 23]^报道了NPE方案与NP方案相比治疗后血小板下降（WHO标准）的情况，分析结果显示NPE方案治疗后重度血小板下降与NP方案相比差异无统计学意义（OR=1.00, 95%CI: 0.64-1.57, *P* > 0.05）（图 5）。2项研究^[16, 21]^报道了NPE+放疗方案与NP+放疗方案相比治疗后血小板下降（WHO标准）的情况，分析结果显示NPE+RT方案治疗后血小板下降与NP+RT方案相比差异无统计学意义（OR=0.78, 95%CI: 0.19-3.16）（图 6）。

**5:**
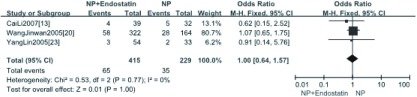
NP+重组人血管内皮抑素方案与NP方案治疗后重度血小板下降*meta*分析 *Meta*-analysis of severe thrombocytopenia between NPE treatment and NP treatment

**6:**
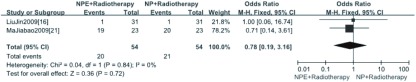
NPE+放疗方案与NP+放疗方案治疗后血小板下降的*meta*分析 *Meta*-analysis of thrombocytopenia after treatment between NPE+RT and NP+RT

##### 2.2.2.3 恶心呕吐

6项研究^[7, 12, 13, 20, 22, 23]^报道了治疗后恶心呕吐发生的例数，分析结果显示NPE方案与NP方案相比恶心呕吐反应差异无统计学意义（OR=0.85, 95%CI: 0.61-1.20, *P* > 0.05）（图 7）。

**7:**
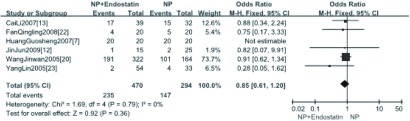
NP+重组人血管内皮抑素方案与NP方案治疗后恶心呕吐的*meta*分析 *Meta*-analysis of nausea and vomiting between NPE and NP scheme

##### 2.2.2.4 放射性食管炎

2项研究^[16, 21]^报道了治疗后放射性食管炎发生的例数，分析结果显示NPE+RT方案与NP+RT方案相比发生放射性食管炎的差异无统计学意义（OR=1.00, 95%CI: 0.40-2.49）（图 8）。

**8:**
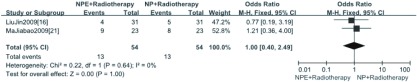
NPE+放疗方案与NP+放疗方案治疗后发生放射性食管炎的*meta*分析 *Meta*-analysis of radioactive esophagitis after treatment between NPE+RT and NP+RT

#### 2.2.3 生存情况

##### 2.2.3.1 中位肿瘤进展时间

4项研究^[12, 18, 20, 23]^报道了中位肿瘤进展时间，NPE方案和NP方案的中位肿瘤进展时间分别为6.3个月和3.6个月、146.68天和91.12天、6.3个月和3.6个月（*P* < 0.000, 1）、151天和100天（*P* < 0.001）。

##### 2.2.3.2 1年无进展生存率、1年生存率、生存质量及治疗相关性死亡

1项研究^[21]^报道了治疗后1年无进展生存率、1年生存率、生存质量的情况，故无法进行定量*meta*分析，可进行描述性分析。该研究共纳入46例确诊的晚期NSCLC患者，试验组与对照组的RR相比无统计学差异（*P*=0.326）。试验组和对照组1年的生存率分别为74.1%（17/23）和65.4%（15/23），1年无进展生存率分别为56.7%（13/23）和52.3%（12/23），试验组1年无进展生存率和1年生存率均高于对照组，但没有统计学差异（*P* > 0.05）。试验组和对照组生存质量均有不同程度的改善，但差异无统计学意义（*P*=0.681）。1项研究^[20]^报道了5例治疗相关性死亡，其中试验组有3例，死亡原因为剧烈腹痛、骨髓抑制引起的严重感染；对照组有2例，死亡原因为严重感染和呼吸功能衰竭。

#### 2.2.4 NOE方案与NO方案、GPE方案与GP方案、GPE方案与T方案、TCE方案与TC方案、TPE方案与TP方案、TE方案与T方案比较

只有1篇文献对相应方案进行了比较，故无法进行*meta*分析。6项研究^[8-11, 14, 15]^在不同干预措施处理各组后，均对RR进行了报道并进行了统计学分析，同时对不良反应也进行了报道，见表 4。

**4:** 不同方案处理后处理组与对照组在RR、TTP及不良反应方面的比较 Comparison of RR, TTP and adverse response between treatment arm and control group

Study	Invention	RR	TTP (month)	Leukopenia	Reduced hemoglobin	Thrombocytopenia	Nausea, vomit	Treatment-related death
Chen SJ^[10]^	NOE/NO	*P* < 0.05	6.6/3.7	*P* > 0.05	*P* > 0.05	*P* > 0.05	*P* > 0.05	none
Xie YR^[9]^	GPE/GP	*P* > 0.05	7/4.5	*P* > 0.05	*P* > 0.05	*P* > 0.05	*P* > 0.05	none
Zhang T^[14]^	GPE/T	*P* < 0.05	Not reported	*P* > 0.05	Not reported	*P* > 0.05	*P* > 0.05	not reported
Tang Z^[8]^	TCE/TC	*P* < 0.05	Not reported	*P* > 0.05	Not reported	Not reported	*P* > 0.05	not reported
Han LC^[11]^	TPE/TP	*P* < 0.05	Not reported	*P* > 0.05	*P* > 0.05	*P* > 0.05	*P* > 0.05	none
Liu J^[15]^	TE/T	*P* < 0.05	Not reported	*P* > 0.05	*P* > 0.05	*P* > 0.05	*P* > 0.05	not reported

## 3 讨论

本文共纳入15项研究，均为临床随机对照研究，全为中文。15研究都进行了相关化疗方案的比较，其中7项研究^[7, 12, 13, 18, 20, 22, 23]^进行了NPE方案与NP方案的比较，2项研究^[16, 21]^进行了NPE+放疗方案与NP+放疗方案的比较。

纳入的15项研究均可能发生中度偏倚。由于肿瘤内科治疗的特性，难以充分采用随机和盲法及对随机进行隐藏，因此纳入的研究所具有的偏倚对于药物治疗肿瘤的临床研究而言可以接受。

### 3.1 NPE方案与NP方案比较

NP方案是目前NSCLC治疗中有效率最高的一线标准方案之一，共纳入831例患者的*meta*分析表明NPE方案与NP方案的RR存在统计学差异。纳入不同例数患者的*meta*分析显示NPE方案与NP方案致治疗后重度白细胞下降、重度血小板下降、恶心呕吐发生的差异均无统计学意义。1项研究报道发生了治疗相关性死亡，其中试验组有3例，死亡原因为剧烈腹痛、骨髓抑制引起的严重感染；对照组有2例，死亡原因为严重感染和呼吸功能衰竭。3项研究报道了采用ECGO评分或karnofsky评分的生存质量评分，分别提示试验组和对照组差异无统计学意义，3项研究均报道了*P*值。1项研究对影响两组患者中位肿瘤进展时间的因素进行了比较，结果表明试验组与对照组相比男性、年龄≥40岁、ECGO评分0分-1分、临床分期为Ⅳ期、鳞癌和腺癌、器官转移数1个-2个以及初治和复治的患者中，RR有统计学差异。纳入的7项研究均未报道1年生存率。与NP方案相比，NPE方案在晚期NSCLC治疗中可以提高近期有效率，同时不良反应的发生并不明显增加，这些研究均没有远期疗效方面的报道，对总生存率是否有优势仍不清楚，纳入的研究结论也不尽一致，仍需要大样本的临床研究验证NPE在反应率上的优势。

### 3.2 NPE+放疗方案与NP+放疗方案比较

2项研究进行了NPE+放疗方案与NP+放疗方案的比较，*meta*分析共纳入了108例晚期NSCLC患者，结果表明NPE+放疗方案组与NP+放疗方案组治疗后的RR相似，两组治疗后白细胞下降、血小板下降、放射性食管炎发生率相似。1项研究报道了治疗后1年无进展生存率、1年生存率、生存质量的情况，试验组1年无进展生存率和1年生存率均高于对照组但无统计学差异，试验组和对照组生存质量均有不同程度的改善但无统计学差异（*P*=0.681）。NPE+放疗方案治疗晚期NSCLC的近期疗效指标与NP+放疗方案相似，毒副作用相似。由于此分析纳入的晚期NSCLC病例数较少，且文献质量偏低，偏倚发生的可能性较大，尚需更多临床随机对照研究来提供充足的证据支持。

### 3.3 NOE方案与NO方案、GPE方案与GP方案、GPE方案与T方案、TCE方案与TC方案、TPE方案与TP方案、TE方案与T方案比较

均只有1项研究对上述六种方案进行了比较，故无法进行*meta*分析。除GPE方案与GP方案的研究中两组RR无统计学差异外，其余5种方案的RR均具有差异。6种方案在发生恶心呕吐、白细胞降低、血小板降低、脱发、乏力等不良反应方面均无统计学差异，提示NOE方案与NO方案、GPE方案与T方案、TCE方案与TC方案、TPE方案与TP方案、TE方案与T方案在治疗后反应率上前者与后者相比有差异，且不会使常见不良反应的发生增加。这种反应率上的优势可能与抗血管生成药物治疗后，肿瘤血管的正常化时间窗出现，使化疗药物可以更多的进入瘤体内发生作用，同时也拮抗由化疗所引起的肿瘤部位血管生成有关，然而这种优势是否具有临床意义需要更多的证据支持。

## 4 结论

在当前临床研究的基础上铂类与第三代细胞毒药物的联合化疗被认为在晚期NSCLC治疗中是标准的一线化疗方案。随着靶向药物研究的快速进展，近年来已出现一些针对NSCLC的特异性分子靶向药物，分子靶向治疗联合常规肿瘤治疗方案也成为研究的热点之一。重组人血管内皮抑素通过特异性作用于内皮细胞，尤其是微血管内皮细胞，达到阻止内皮细胞迁移并诱导其凋亡而发挥抗血管生成作用。在临床一线治疗中，重组人血管内皮抑素与NP方案联合应用后可以提高近期疗效并且严重毒副反应的发生并不明显增加，而重组人血管内皮抑素联合NP及放疗并没有提高疗效，其毒副反应发生的情况相似。以铂类药物为基础联合重组人血管内皮抑素和紫杉醇类药物与单药相比可以提高近期疗效而不明显增加毒副反应，进一步说明晚期NSCLC的治疗应采取多药联合治疗方案。

纳入的15项研究均为随机对照试验，但采用合理分配隐藏和盲法的研究很少，14项研究没有进行长期随访，具有高度的选择性偏倚和测量偏倚，此外研究的样本数也较少，因此对于试验的结果评价应谨慎。目前肿瘤临床试验对盲法的要求比较低，客观反应率和生存时间是比较客观的衡量指标，以后的研究在方法学上应采用多中心、完全随机并进行匹配随机的研究方法，尽量实施分配隐藏或盲法，力求提高研究报告的质量。还应对样本长期随访，设置并报道终点指标，以及按有关标准充分报道毒副反应。

因此，为了给临床医生提供更合理的临床决策，为癌症患者治疗提供更有效的选择，尚需更多高质量的临床随机对照试验提供高质量的临床证据。
